# Current-Induced Changes of Surface Morphology in Printed Ag Thin Wires

**DOI:** 10.3390/ma12203288

**Published:** 2019-10-10

**Authors:** Quan Sun, Yebo Lu, Chengli Tang, Haijun Song, Chao Li, Chuncheng Zuo

**Affiliations:** 1 College of Mechanical and Electrical Engineering, Jiaxing University, 118 Jiahang Road, Jiaxing 314001, China; 2 School of Mechanical and Power Engineering, East China University of Science and Technology, Shanghai 200237, China

**Keywords:** flexible electronics, electromigration, thin films, joule heating, numerical simulation

## Abstract

Current-induced changes of surface morphology in printed Ag thin wires were investigated by current stressing tests and numerical simulation. The samples were printed Ag thin wires on a flexible substrate with input and output pads. Different experimentalresults were obtainedthroughchangingthe current density after current supply and the mechanism of those phenomena were investigated by numerical simulations based on the method of atomic flux divergence. Good agreement between the simulations and experimental results was reached. It was found that electromigration was the main factor that caused the change of the surface morphology. The contribution of thermal migration can be ignored, and the Joule heating lead by the supplied current had a very significant accelerating effect on electromigration. Guidelines for effectively changing the Ag thin wire surface through providing predetermined current density was proposed, which were expected to be useful for improving the electrical reliability and lifetime of printed Ag thin wires in flexible electronic devices.

## 1. Introduction

Printed flexible electronics including sensors, wearable devices, antennas and electrodes, have become increasingly attractive and important components in the next-generation electronics due to their unique advantages in the physical properties and fabrication process [1,2,3,4,5]. Currently, Ag is the commonly used component material, which is printed as circuits on a flexible substrate, because of its excellent electrical conductivity and mature printing technology [6,7,8]. It is a significant issue to improve the mechanical and electrical reliability of Ag lines in flexible devices, in which current-induced changes of surface morphology cannot be ignored [9,10].

Electromigration (EM), thermal migration (TM) and Joule heating are main reasons for surface change of the Ag line during current supply, and much research has been carried out [11,12,13]. EM, TM and Joule heating usually interact and influence each other; among them EM is a physical phenomenon in which metallic atoms migrate due to electron wind during current supply, which is considered as a critical factor in bringing failure in semiconductor devices. TM is another physical phenomenon in which atomic diffusion occurs due to the thermal gradient caused by Joule heating. Basaran et al. [14,15] reported that both EM and TM could cause material degradation in next-generation nanoelectronics. Hauder et al. [16] investigated EM failure for thin sputtered Ag films and microstructured Ag lines, and the activation energy for EM was found to be 0.58 eV by considering Joule heating of the lines. Chatterjee et al. [17] used EM as a structuring tool in Ag nanowires induced by critical current densities, and voids and hillocks were formed. The Ag wires were fabricated in a vacuum by using pure Ag targets with traditional semiconductor processes in the mentioned research. Very fewdefects existed in these Ag wires, and the atoms diffused mainly along grain boundaries or interface. However, defects such as voids and impurities existed, causing different atomic diffusion path and activation energy in the printed Ag wires. Jang et al. [18] reported particle growth and resistance increase in printed Ag interconnects after current supply due to Joule heating and EM, which eventually resulted in electrical failure. However, the guidelines for controlling current-induced surface morphology to improve reliability were not mentioned.

In the present paper, current-induced changes of surface morphology in printed Ag thin wires were investigated. The experimental samples of printed Ag wires on a flexible substrate were tested, and it was found that the current density significantly affected the wire surface due to atomic diffusion. The mechanism was investigated by finite element simulation based on the method of atomic flux divergence. Guidelines for effectively controlling the atomic diffusion in the printed Ag wire were drawn up. Based on the guidelines, the electrical property of the Ag wire can be improved, and the current-induced failure can also be avoided.

## 2. Experimental Procedures and Results

**Figure 1:**
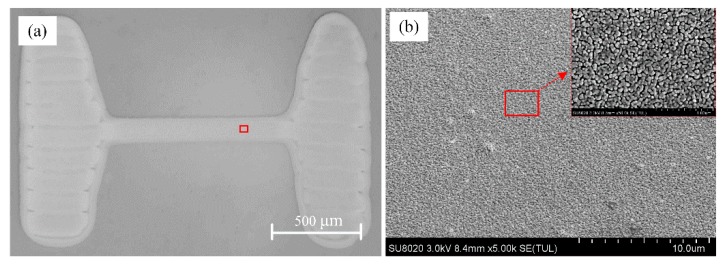
Graph of printed silver wire structure by (**a**) optical microscope and (**b**) local SEM magnification.

A polyethylene terephthalate (PET) substrate with thickness of 120 μm was used as the flexible substrate because of its good flexibility, high light transmission and low cost, and a dog-bone type Ag thin wire was printed using the Ag conductive ink with input and output pads by an inkjet printer (MP1100, Prtronic, Shanghai, China), and the wire thickness and width were controlled to be 100 nm and 100 μm, respectively. After that, the samples were sintered by heating in a furnace at 120 °C for 30 min. The thickness of the Ag film layer was confirmed by using a profile system (DektakXT, Bruker, Germany). A micrograph of sample taken by the microscope was shown in Figure 1a, and a local magnification by field-emission scanning electron microscopy (FE-SEM, SU8020, Hitachi, Tokyo, Japan) shown in Figure 1b indicated that the silver wire was constructed by silver nanoparticles with the main particle size of ~50 nm. The initial resistance of the silver wire measured by the multimeter was 31 ± 0.5 Ω.

The sample was then subjected to a constant direct current in the air at room temperature to perform testing. The testing time was controlled to be less than 1 h in order to protect the sample from bursting as a result of Joule heating. During current supply, the potential drop along the Ag wire was measured between the input and output pads.

**Figure 2:**
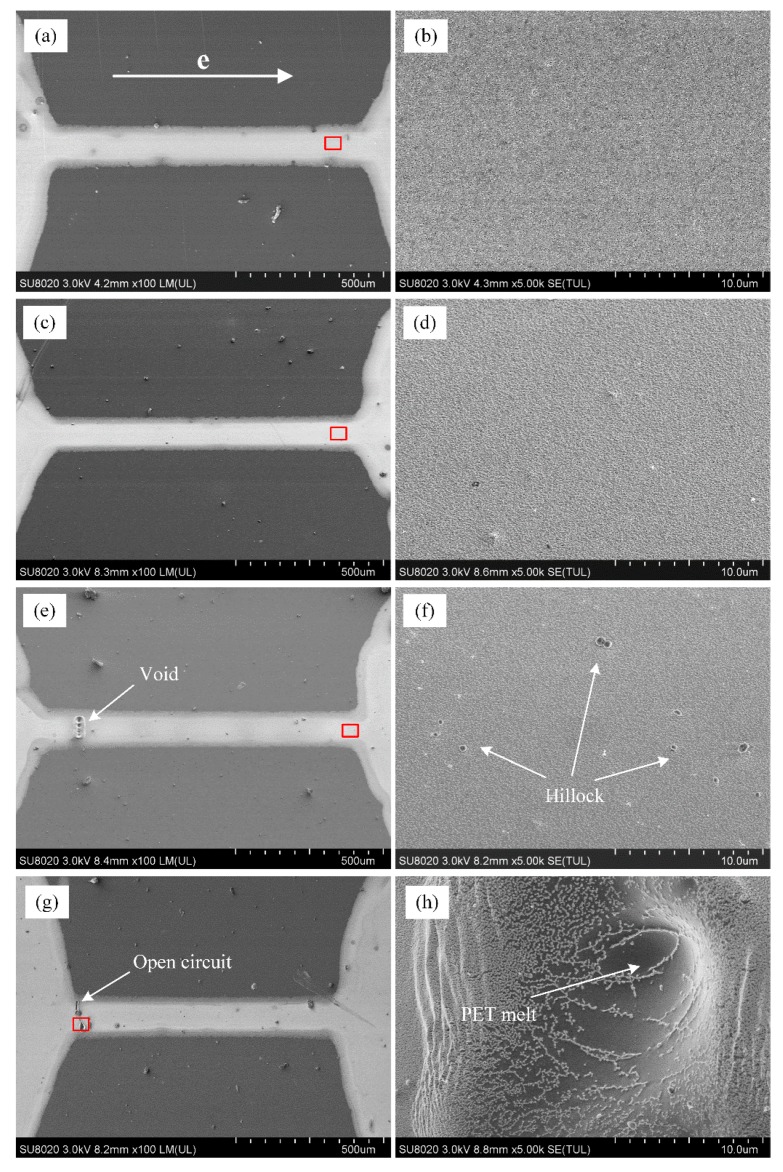
Surface morphology change of printed silver wires after testing with current density of (**a**,**b**) 0.4 MA/cm^2^; (**c**,**d**) 0.8 MA/cm^2^; (**e**,**f**) 1.0 MA/cm^2^; (**g**,**h**) 1.2 MA/cm^2^.

**Figure 3:**
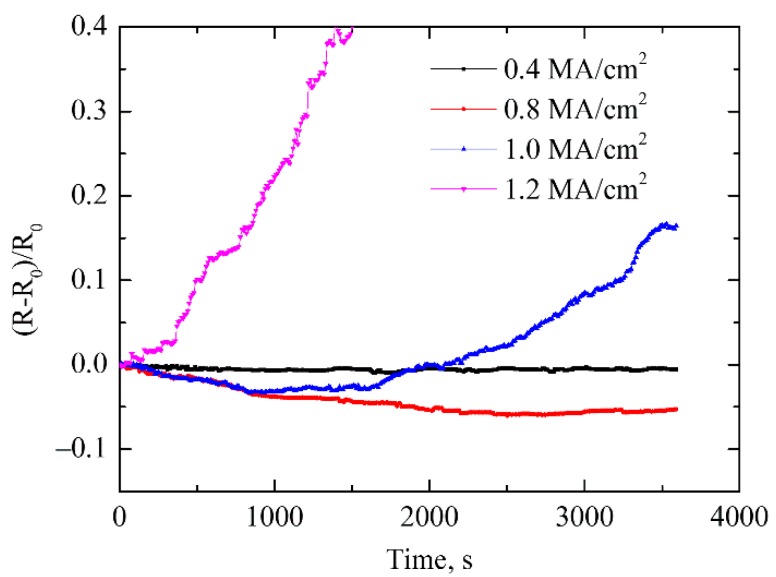
Resistance change of dog-bone type sample during electromigration (EM) testing with different current density.

The experimental conditions and results for four examples of samples I–IV are shown in Table 1. Ten samples were prepared for each current density. Under repeatability conditions, similar experimental results were obtained. After current supply, the surfaces of samples were observed as shown in Figure 2, and the corresponding relationships between the potential drop and the current supply time are shown in Figure 3. The sample surface and the electrical resistance had little change in sample I, indicating the current density was not large enough to cause mass atomic diffusion. The electrical resistance dropped obviously at the initial stage and then gradually leveled off in sampleII, and the surface microstructure became denser than the initial state. In sample III, the electrical resistance change curve showed a trend of first decline and then rapid increase, and the microstructure became denser as the same with sample II, however, voids and hillocks were observed in the silver thin wire near the input and output pads. In sample IV, the electrical resistance increased sharply until open circuit and breakage was observed in the silver wire near the output pad.

## 3. Atomic Migration and Simulation

Let us consider the atomic diffusion in the printed thin wire. As the silver wires were conducted under accelerated conditions with very large current density in the test, and the Ag atoms were migrated in a specified direction due to electron wind. At the same time, high density current could lead to local Joule heating and form temperature gradients in the silver wire, which would cause TM in the silver wire.

The atomic diffusion is governed by the mass conservation equation:

(1) $$\mathrm{div}\left(q\right)+\frac{\partial c}{\partial t}=0$$

where *c* is the normalized atomic concentration, *c = C/C*_0_, *C* is the actual atomic concentration, *C*_0_ is the initial atomic concentration in the absence of a stress field, *t* is the time and *q* is the total normalized atomic flux.

Considering that the driving forces of atomic flux include the electron wind force, thermal gradient and atomic concentration gradient, thus the total normalized atomic flux can be written as:

(2) $$q=q_{EM}+q_{TM}+q_{C}=\frac{cD}{k_{B}T}Z^{*}e\rho j-\frac{cD}{k_{B}T}Q^{*}\frac{\nabla T}{T}-D\nabla c$$

where *D* is the effective atom diffusivity, $D=D_{0}\exp\left[-E_{a}/\left(k_{B}T\right)\right]$, $E_{a}$ is the activation energy and $D_{0}$ is the effective thermally activated diffusion coefficient, $k_{B}$ is the Boltzmann’s constant, T is the absolute temperature, e is the electronic charge, $Z^{*}$ is the effective charge, $Q^{*}$ is the heat capacity per atom, j is the current density vector, ρ is the resistivity which is calculated as $\rho =\rho_{0}\left[1+\alpha \left(T-T_{0}\right)\right]$, where α is the temperature coefficient of the metallic material and $\rho_{0}$ is the resistivity at $T_{0}$.

To locate the position of voids and hillocks, the atomic flux divergence (AFD) was calculated. The value of AFD represents the number of atoms increasing (AFD takes negative value) or decreasing (AFD takes positive value) per unit volume and unit time for hillock or void formation. According to Equation (2), the divergence of total normalized atomic flux can be written as

(3) $$\begin{array}{ll} \mathrm{div}\left(q\right) & =\left(\frac{E_{a}}{k_{B}T^{2}}+\alpha \frac{\rho_{0}}{\rho }-\frac{1}{T}\right)q_{EM}\nabla T+\frac{1}{c}q_{EM}\nabla c\\ & +\left(\frac{E_{a}}{k_{B}T^{2}}-\frac{2}{T}\right)q_{TM}\nabla T+\frac{1}{c}q_{TM}\nabla c-\frac{cDQ^{*}}{k_{B}T^{2}}\nabla^{2}T\\ & +\frac{E_{a}}{k_{B}T^{2}}q_{C}-D\nabla^{2}c \end{array}$$

The finite element (FE) program of ABAQUS was used to simulate the atomic diffusion behavior based on the concept of atomic flux divergence. Firstly, the electrical–thermal field coupling simulation of silver wire was conducted to obtain the distribution of current density vector and temperature. Then, the atomic flux divergence of each node of the finite element mesh was calculated iteratively by using ABAQUS/Python (6.14, Simulia Corporation, Vélizy-Villacoublay, France) script and the atomic concentration at a special time was obtained. 

**Figure 4:**
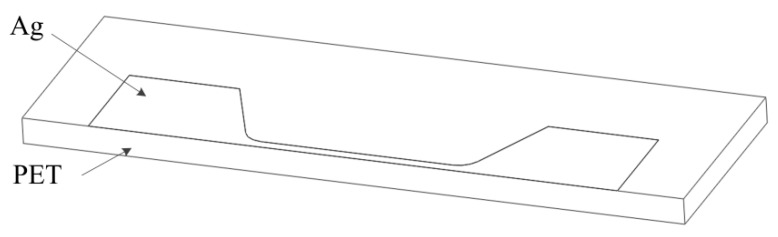
Finite element model of a silver wire sample.

The finite element model of the silver wire sample was shown in Figure 4 and a half FE model was applied. The sizes of the FE model were set as same as the experimental sample with wire thickness of 100 nm and width of 50 μm, and the thickness of PET substrate was 120 μm. For the electrical–thermal coupling simulation, the material parameters of silver and PET are listed in Table 2.

Based on the electrical–thermal coupling simulation results of temperature and current density distribution, the EM and TM were simulated by using ABAQUS/Python script. The EM parameters of Ag were selected from Reference [19] as shown in Table 3.

## 4. Discussion

### 4.1. Joule Heating of Silver Wire under Current Supply

**Figure 5:**
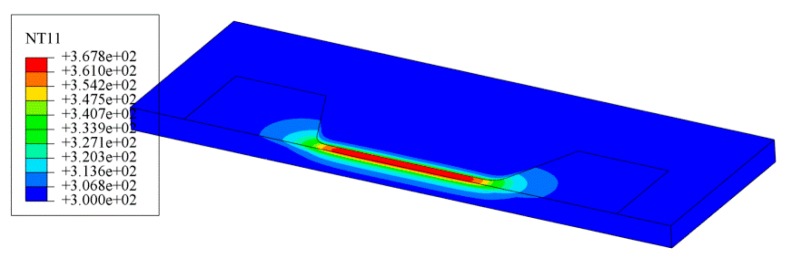
Simulation result of temperature distribution with current density of 0.6 MA/cm^2^.

**Figure 6:**
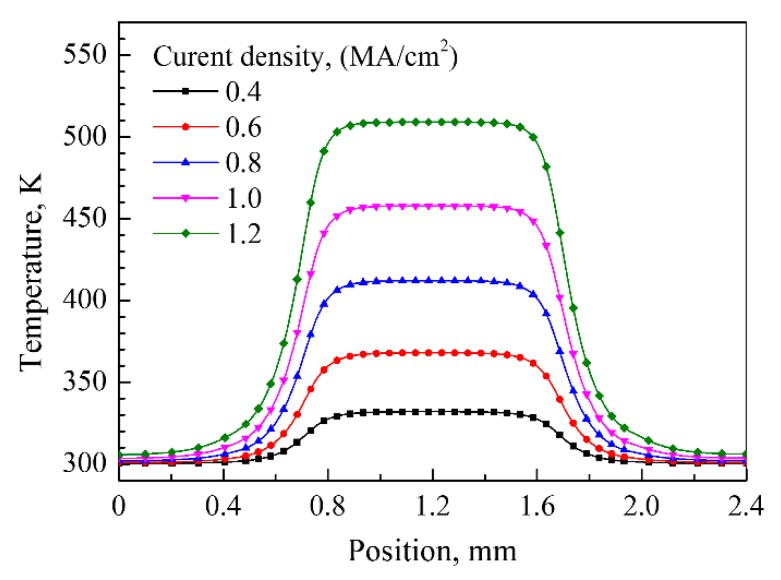
Temperature distribution in silver wire under current density of 0.4~1.2 MA/cm^2^.

The electrical–thermal field coupling simulations of silver wire were conducted under current supply with different current density. The temperature distribution of the silver wire obtained by simulation was shown in Figure 5. The sample temperature increased to 367.8 K due to Joule heating as the room temperature was 300 K when the current density was set to be 0.6 MA/cm^2^, and the temperature in the silver wire took the maximum value at the center of the narrow part and decreased towards the wider part of the structure. This trend was observed for all simulations under different current densities of 0.4~1.2 MA/cm^2^ and the temperature distribution along the silver wire length direction at the center line is shown in Figure 6.

From the results of the electrical–thermal coupling simulations, it can be seen that a large amount of Joule heating was generated during the accelerated EM testing due to the current supply with high density, which caused a significant rise in temperature in the silver wire. The high temperature can promote the formation of more sintering necks between silver nanoparticles and increase the electrical conductivity. The experimental results shown in Figure 3 indicated that the resistances of the silver wires had descent tendency during current supply with current density of 0.6 MA/cm^2^ and 0.8 MA/cm^2^, which can be explained by the sintering effect of Joule heating. The increased temperature of the two samples obtained by simulation were 367.8 K and 411.3 K, respectively, which are close to and over the sintering temperature (120 ℃/393 K) of the printed silver wire.

The temperature of the silver wire increased to be as high as 509 K when the current density was 1.2 MA/cm^2^, which is slightly lower than PET material melt temperature (~260 ℃/533 K). However, during the current supply with high density, the formation of voids by EM and TM could have increased the local current density, which enhanced the Joule heating effect and increased the local temperature and finally reached the melting point of PET and caused the failure of the silver wire.

### 4.2. Atomic Migration of Silver Wire by EM and TM

**Figure 7:**
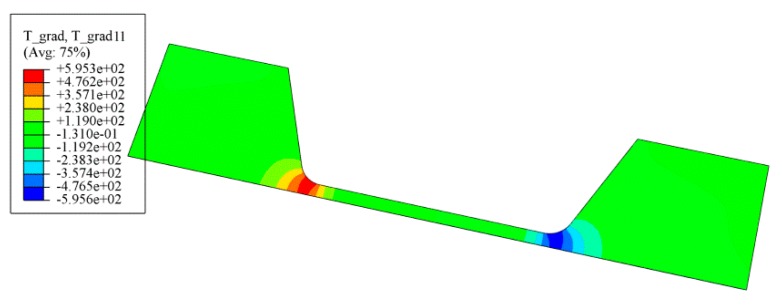
Simulation result of temperature gradient with current density of 0.8 MA/cm^2^.

From the simulation results of temperature distribution of silver wire sample shown in Figure 6, a significant temperature gradient can be observed from the transition area of narrow wire to the wide electrode part, and the gradient increased with the density of current supply. Figure 7 shows the distribution of temperature gradient along the length direction of the silver wire sample with current density of 0.8 MA/cm^2^. The large temperature gradient was an important driving force of the atomic migration in the silver wire, which was known as TM.

**Figure 8:**
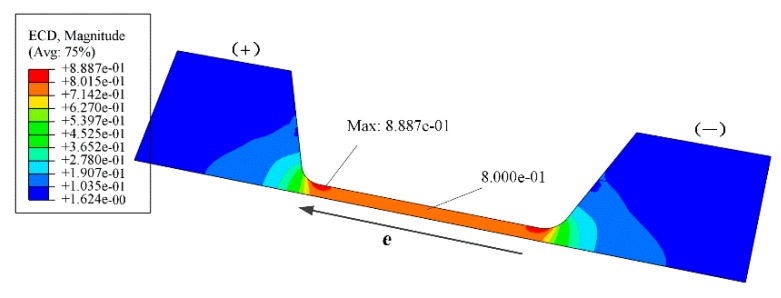
Distribution of the current density in the silver wire structure (unit: MA/cm^2^).

The electron wind formed by high density current was another driving force of the atomic migration. Figure 8 shows the current density distribution in the silver wire. As the center part of narrow wire suffered current density of 0.8 MA/cm^2^, the distribution of current density was not uniformly across the cross section. The maximum current density can reach as high as 0.8887 MA/cm^2^ at the edges where the narrow wire connected to wider part of the structure, which was the vulnerable area of the silver wire in the EM testing.

**Figure 9:**
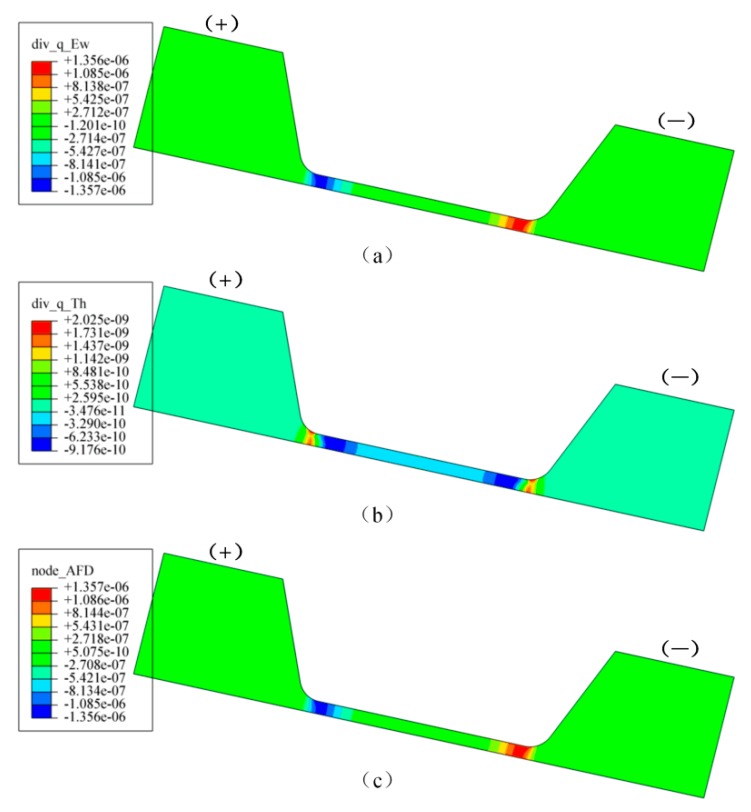
Distribution of atomic flux divergence by (**a**) current, (**b**) temperature gradient and (**c**) total atomic flux divergence (AFD) with current density of 0.8 MA/cm^2^.

The AFD distribution calculated based on the current density and temperature gradient obtained by electrical–thermal coupling simulation result with current density of 0.8 MA/cm^2^ is shown in Figure 9. The AFD caused by current shown in Figure 9a was larger than that caused by temperature shown in Figure 9b by about three orders, therefore, the effect of temperature gradient produced by Joule heating on the atomic migration in the silver wire could be ignored. However, it should be noted that the high temperature due to Joule heating strengthened the effective atom diffusivity significantly and accelerated the atom migration in EM testing. The total AFD distribution without taking into account the atomic concentration gradient in the silver wire is shown in Figure 9c. The maximum value of AFD was located in the area of silver wire close to the two electrode pads. In the region nearby the positive electrode, the AFD value was negative which indicated that Ag atoms would accumulate and form hillocks in this place, and the region of silver wire nearby the negative electrode, the AFD value was positive which implied that the Ag atoms would migrate out and form voids.

**Figure 10:**
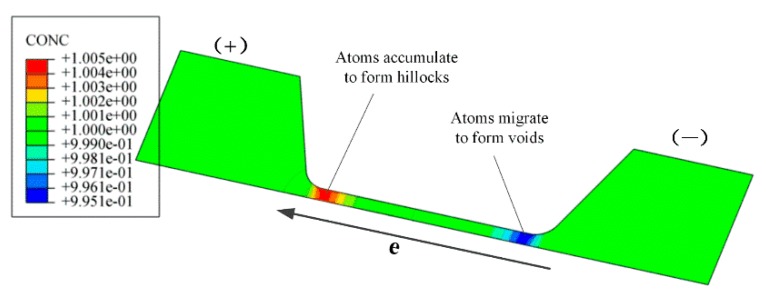
Distribution of normalized atomic concentration after 1 h current supply with density of 0.8 MA/cm^2^.

Figure 10 shows the distribution of normalized concentration in the silver wire sample after 1 h current supply with a density of 0.8 MA/cm^2^. It can be seen that the atoms accumulating in the area close to the positive electrode pad and migrating in the area close to the negative electrode pad will form hillocks and voids, respectively, causing the failure of the silver wire. The simulation result was consistent with the experimental results as shown in Figure 2.

**Figure 11:**
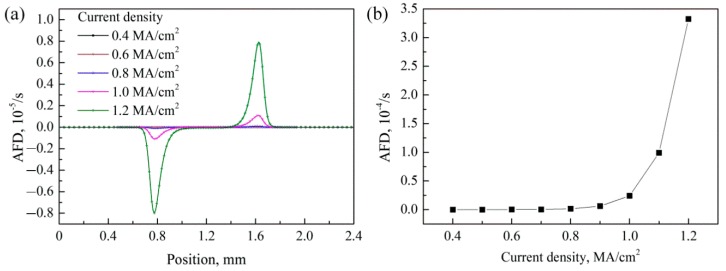
(**a**) AFD distribution in silver wire under current density of 0.4~1.2 MA/cm^2^ and (**b**) the maximum AFD value under different current density.

In order to investigate the EM and TM behavior of silver wire under different current density, the atomic migration behavior of silver wire was simulated with current density of 0.4~0.8 MA/cm^2^. The AFD value along the silver wire with different current density at initial time is shown in Figure 11a, and the maximum AFD value increased exponentially with the current density as shown in Figure 11b.

**Figure 12:**
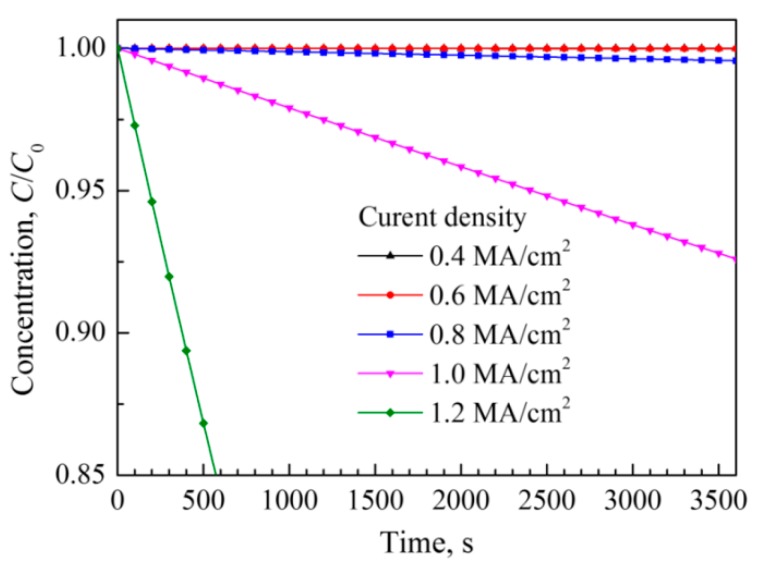
Normalizedatomic concentration in the area nearby the negative electrode pad during current supply with different densities.

In the silver wire, the voids formation by atomic migration was the important factor that affected the electrical properties of silver wire. Figure 12 presents the variation of normalized atomic concentration in the area nearby the negative electrode pad of the silver wire during the accelerated EM testing. It showed that the atomic concentration had little change when the current density was below 0.6 MA/cm^2^ after 1 h testing, however, the atomic concentration increased sharply with the increase of current density.

**Figure 13:**
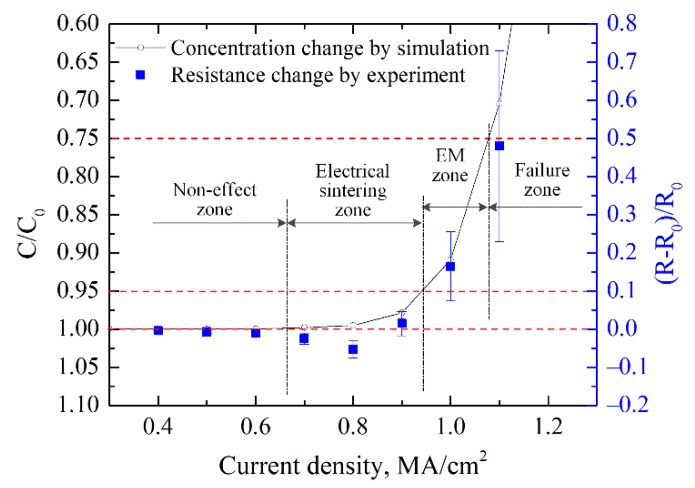
The different behavior of silver wire with current supply for 1 h with different densities.

According to the experimental and simulation results, the different behavior of silver wire on the PET substrate under accelerated EM testing with different current density is illustrated in Figure 13. The electrical resistance and surface morphology kept almost unchanged when the current density was below 0.63 MA/cm^2^. However, when the current density was larger, the Joule heating in the silver wire caused the temperature to rise by 100 ℃ which could sinter the silver wire and decreased the electrical resistance. Meanwhile, higher current density led to a significant EM phenomenon and caused failure of the silver wire. It was assumedthat no void formed in the silver wire when the normalized concentration was higher than 0.95 with the current density of ≤0.93 MA/cm^2^. Voids and hillocks were formed when the normalized concentration was lower than 0.75 with current density of ≥1.1 MA/cm^2^, causing an increase of local current density and acceleration of the silver wire damage, and finally breakage failure occurred in the silver wire. Thus, the current density from 0.63 MA/cm^2^ to 0.93 MA/cm^2^ can be defined as electrical sintering zone which can improve the electrical conductivity of silver wire during the current supply. A large number of voids and hillocks formed and led to the increase of silver wire electrical resistance when the current density was 0.93~1.1 MA/cm^2^ which could be named as the EM zone. When the current density was larger than 1.1 MA/cm^2^, an open circuit occurred in the silver wire because of atomic diffusion of EM and TM or PET substrate melting due to Joule heating.

## 5. Conclusions

The electrical sintering and atomic diffusion behavior of the printed Ag thin wire under different current density was investigated by experiments and numerical simulation. Four different experimental results were observed in the accelerated testing. The morphology of the silver wire was unchanged when the current density was below 0.63 MA/cm^2^. The electrical conductivity of the silver wire increased when current density was 0.63~0.93 MA/cm^2^ because of electrical sintering of Joule heating, and decreased with current density of 0.93~1.1 MA/cm^2^ because of atomic diffusion by EM and TM. An open circuit occurred in the Ag wire due to mass atomic migration or melting of the PET substrate by Joule heating when the current density was larger than 1.1 MA/cm^2^. The results were expected to be guidelines for improving the electrical property and enhancing lifetime of the printed Ag thin wires in flexible electronic devices.

## Figures and Tables

**Table 1:** Experimental conditions and results of four cases with different current density.

Samples	Current Density (MA/cm^2^)	Current Supply Time (h)	Resistance Change	Surface Morphology
I	0.4	1	−0.4%	Unchanged
II	0.8	1	−5.3%	Densification
III	1.0	1	16.5%	Voids and hillocks
IV	1.2	less than 1	+∞	Open circuit

**Table 2:** Material parameters used in the electrical–thermal coupling simulation.

Parameters	Unit	Ag	PET
Density	g/cm^3^	10.49	1.33
Young’s Modulus	GPa	73.2	4
Poisson’s Ratio	-	0.38	0.3
Thermal Conductivity	W/(m·K)	429	0.2
Electrical Conductivity	S/m	6.3 × 10^7^	0
Specific Heat	J/(g·K)	240	1200
Emissivity	-	0.04	0.8

**Table 3:** EM parameters of Ag used in simulation.

Parameters	Value
*E*_a_	0.9 eV
*D*_0_	1.71 × 10^−5^ m^2^/s
Z*	−21
Q*	−0.0867 eV
Ω	1.71 ×10^−29^ m^3^/atom
*ρ*_0_	1.6 × 10^−7^ Ω·m
*α*	3.8 × 10^−3^ 1/K
